# Supplementary material for: COVID-19 and vaccine hesitancy: A longitudinal study
Source: PLoS One. 2021 Apr 16;16(4):e0250123. doi: 10.1371/journal.pone.0250123 (PMC8051771; doi:10.1371/journal.pone.0250123)
Supplement: S1 Appendix — (DOCX) [file pone.0250123.s001.docx]

**S1 Appendix. Additional information about sample exclusions.** 407 participants responded to all 6 waves (waves 1, 2, 3, 4, 5, and 6 had 1,018, 762, 654, 608, 652, and 667 participants, respectively). If a participant responded to wave 1 multiple times, they were removed (N = 15). If they responded to waves 2, 3, 4, 5, or 6 multiple times, their observations for that wave were removed (Ns = 4, 8, 57, 4, 10, respectively). Seven observations were removed due to a missing Amazon MTurk ID. These exclusions represent 2.4% of observations.
