# Supplementary material for: COVID-19 and vaccine hesitancy: A longitudinal study
Source: PLoS One. 2021 Apr 16;16(4):e0250123. doi: 10.1371/journal.pone.0250123 (PMC8051771; doi:10.1371/journal.pone.0250123)
Supplement: S2 Appendix — (DOCX) [file pone.0250123.s002.docx]

**S2 Appendix. Additional information about political party affiliation.** Political affiliation correlated with participants’ age. Among participants in the first wave, Republicans were, on average, 3.6 years older than Democrats (*M*_D_ = 37.14, SD_D_ = 11.86; *M*_R_ = 40.7, SD_R_ = 12.47; *t* = 4.46, *p* < .001). Republicans also reported higher subjective socioeconomic status (MacArthur Scale of subjective SES; [49]) than Democrats (*M*_D_ = 4.82, SD_D_ = 1.60; *M*_R_ = 5.15, SD_R_ = 1.54; *t* = 2.89, *p* = .004). Gender identity did not significantly differ by political affiliation (*p* > .36).
