# Supplementary material for: COVID-19 and vaccine hesitancy: A longitudinal study
Source: PLoS One. 2021 Apr 16;16(4):e0250123. doi: 10.1371/journal.pone.0250123 (PMC8051771; doi:10.1371/journal.pone.0250123)
Supplement: S1 Table — (DOCX) [file pone.0250123.s003.docx]

**S1 Table. Attrition table.**

| **Variable** | **Wave Participants** | **Wave 1 mean (sd)** | **N** |
| --- | --- | --- | --- |
| Age | 1 | 38.48 (12.21) | 997 |
|  | 2 | 39.93 (12.54)* | 762 |
|  | 3 | 40.59 (12.49)*** | 654 |
|  | 4 | 40.80 (12.43)*** | 608 |
|  | 5 | 40.14 (12.47)** | 651 |
|  | 6 | 40.33 (12.39)** | 666 |
| Gender (female) | 1 | 46.19% | 461 |
|  | 2 | 46.19% | 352 |
|  | 3 | 46.02% | 301 |
|  | 4 | 45.72% | 278 |
|  | 5 | 45.86% | 299 |
|  | 6 | 47.08% | 314 |
| Political Party | 1 | 3.98 (1.49) | 998 |
|  | 2 | 3.95 (1.50) | 762 |
|  | 3 | 4.00 (1.50) | 654 |
|  | 4 | 3.95 (1.52) | 608 |
|  | 5 | 3.97 (1.52) | 652 |
|  | 6 | 4.00 (1.49) | 667 |
| COVID-19 Vaccination Attitudes | 1 | 5.08 (1.68) | 1004 |
|  | 2 | 5.04 (1.72) | 762 |
|  | 3 | 5.06 (1.70) | 654 |
|  | 4 | 5.06 (1.68) | 608 |
|  | 5 | 5.05 (1.72) | 652 |
|  | 6 | 5.06 (1.70) | 667 |
| General Vaccination Attitudes | 1 | 5.59 (1.25) | 972 |
|  | 2 | 5.61 (1.26) | 739 |
|  | 3 | 5.64 (1.23) | 636 |
|  | 4 | 5.63 (1.23) | 589 |
|  | 5 | 5.65 (1.27) | 631 |
|  | 6 | 5.64 (1.27) | 645 |
| Flu Shot Intentions | 1 | 4.66 (2.37) | 992 |
|  | 2 | 4.69 (2.37) | 759 |
|  | 3 | 4.71 (2.40) | 651 |
|  | 4 | 4.71 (2.40) | 605 |
|  | 5 | 4.72 (2.39) | 652 |
|  | 6 | 4.66 (2.42) | 665 |
| Perceived Threat of COVID-19 | 1 | 4.13 (1.31) | 999 |
|  | 2 | 4.06 (1.31) | 757 |
|  | 3 | 4.06 (1.34) | 650 |
|  | 4 | 4.11 (1.29) | 604 |
|  | 5 | 4.05 (1.32) | 648 |
|  | 6 | 4.10 (1.30) | 663 |
| Trust: Media | 1 | 3.28 (1.70) | 1003 |
|  | 2 | 3.24 (1.68) | 761 |
|  | 3 | 3.27 (1.69) | 653 |
|  | 4 | 3.31 (1.71) | 608 |
|  | 5 | 3.29 (1.71) | 651 |
|  | 6 | 3.31 (1.72) | 666 |
| Trust: Local Government | 1 | 4.15 (1.61) | 1003 |
|  | 2 | 4.18 (1.61) | 761 |
|  | 3 | 4.17 (1.59) | 653 |
|  | 4 | 4.26 (1.59) | 608 |
|  | 5 | 4.20 (1.59) | 651 |
|  | 6 | 4.19 (1.58) | 666 |
| Trust: Federal Government | 1 | 3.38 (1.73) | 1003 |
|  | 2 | 3.38 (1.72) | 761 |
|  | 3 | 3.37 (1.68) | 653 |
|  | 4 | 3.43 (1.74) | 608 |
|  | 5 | 3.36 (1.72) | 651 |
|  | 6 | 3.34 (1.72) | 666 |

Significance codes: *** P < .001, ** P < .01, * P < .05.

S1 Table shows the wave 1 characteristics for participants who responded to each wave. For gender, only the proportion of females is shown; participants identifying as “another gender” constituted around 0.65% of the sample in each wave. For all variables, we tested whether participants who responded to waves 2-6 were significantly different at baseline (wave 1) from the full sample at baseline (chi-squared test for gender, t-test for all others).
