# Supplementary material for: COVID-19 and vaccine hesitancy: A longitudinal study
Source: PLoS One. 2021 Apr 16;16(4):e0250123. doi: 10.1371/journal.pone.0250123 (PMC8051771; doi:10.1371/journal.pone.0250123)
Supplement: S2 Table — (DOCX) [file pone.0250123.s004.docx]

**S2 Table. Summary table of measures and constructs included in the text.**

| **Measures and Constructs** | **Scale Anchors** | **Mar (W1)** | **Apr (W2)** | **May (W3)** | **Jun (W4)** | **Jul (W5)** | **Aug (W6)** |
| --- | --- | --- | --- | --- | --- | --- | --- |
| COVID-19 VACCINATION ATTITUDES (CONSTRUCT) |  | 5.08 (1.68) | 5.24 (1.77) | 5.07 (1.88) | 4.99 (1.90) | 5.06 (1.91) | 4.84 (1.97) |
| When there is a vaccine available for the Coronavirus, I will get it. | 1 = strongly disagree, 7 = strongly agree | 5.25 (1.66) | 5.52 (1.71) | 5.43 (1.82) | 5.35 (1.86) | 5.37 (1.88) | 5.21 (1.93) |
| When there is a vaccine for the Coronavirus, it should be mandatory. | 1 = strongly disagree, 7 = strongly agree | 4.91 (1.92) | 4.95 (2.05) | 4.70 (2.15) | 4.63 (2.14) | 4.75 (2.17) | 4.47 (2.22) |
| GENERAL VACCINATION ATTITUDES (CONSTRUCT) |  | 5.59 (1.25) | 5.56 (1.29) | 5.55 (1.27) | 5.54 (1.32) | 5.57 (1.32) | 5.50 (1.35) |
| Vaccines are safe | 1 = strongly disagree, 7 = strongly agree | 5.78 (1.34) | 5.65 (1.42) | 5.70 (1.37) | 5.61 (1.44) | 5.68 (1.38) | 5.57 (1.48) |
| Vaccines contain dangerous ingredients® | 1 = strongly disagree, 7 = strongly agree | 4.98 (1.81) | 5.08 (1.75) | 5.04 (1.74) | 5.08 (1.78) | 5.06 (1.81) | 5.06 (1.79) |
| Some vaccines are unnecessary since they target relatively harmless diseases® | 1 = strongly disagree, 7 = strongly agree | 5.18 (1.83) | 5.28 (1.77) | 5.20 (1.78) | 5.26 (1.78) | 5.24 (1.83) | 5.17 (1.80) |
| Vaccines are effective at preventing disease | 1 = strongly disagree, 7 = strongly agree | 6.06 (1.18) | 6.06 (1.13) | 6.04 (1.14) | 6.03 (1.17) | 6.04 (1.18) | 5.95 (1.24) |
| We give children the right number of vaccines | 1 = strongly disagree, 7 = strongly agree | 5.36 (1.49) | 5.38 (1.49) | 5.36 (1.46) | 5.34 (1.54) | 5.39 (1.53) | 5.24 (1.62) |
| We give children too many vaccines® | 1 = strongly disagree, 7 = strongly agree | 5.30 (1.82) | 5.27 (1.82) | 5.33 (1.75) | 5.28 (1.83) | 5.34 (1.77) | 5.29 (1.79) |
| Vaccines conflict with my belief that children should use natural products and avoid toxins® | 1 = strongly disagree, 7 = strongly agree | 5.69 (1.71) | 5.67 (1.75) | 5.71 (1.64) | 5.73 (1.69) | 5.79 (1.65) | 5.72 (1.66) |
| Vaccines are a major advancement for humanity | 1 = strongly disagree, 7 = strongly agree | 6.11 (1.16) | 6.08 (1.25) | 6.10 (1.22) | 6.09 (1.21) | 6.13 (1.21) | 6.09 (1.28) |
| The government should not force children to get vaccinated to attend school® | 1 = strongly disagree, 7 = strongly agree | 5.40 (1.86) | 5.28 (1.89) | 5.26 (1.85) | 5.28 (1.92) | 5.28 (1.88) | 5.24 (1.90) |
| To protect public health we should follow government guidelines about vaccines | 1 = strongly disagree, 7 = strongly agree | 5.88 (1.30) | 5.83 (1.37) | 5.76 (1.33) | 5.71 (1.44) | 5.75 (1.39) | 5.65 (1.52) |
| FLU SHOT INTENTIONS: Do you plan on getting the flu vaccine next year? | 1 = definitely no, 7 = definitely yes | 4.66 (2.37) | 4.70 (2.38) | 4.67 (2.40) | 4.57 (2.40) | 4.67 (2.44) | 4.58 (2.44) |
| PERCEIVED THREAT OF COVID-19 (CONSTRUCT) |  | 4.13 (1.31) | 4.32 (1.30) | 4.12 (1.41) | 4.00 (1.42) | 4.23 (1.41) | 4.18 (1.41) |
| How are you currently feeling about the Coronavirus situation? | 1 = it feels like it is not a big deal, 7 = it feels like it is the end of the world | 4.40 (1.37) | 4.60 (1.30) | 4.31 (1.40) | 4.11 (1.45) | 4.49 (1.40) | 4.30 (1.43) |
| To what extent do you feel personally at risk of getting the Coronavirus? | 1 = very little personal risk, 7 = very high personal risk | 3.75 (1.67) | 3.86 (1.69) | 3.73 (1.70) | 3.66 (1.65) | 3.86 (1.73) | 3.87 (1.72) |
| How would you describe your beliefs about what would happen if you contracted the Coronavirus? | 1 = not at all serious, 7 = very serious | 4.22 (1.67) | 4.51 (1.65) | 4.32 (1.73) | 4.24 (1.76) | 4.32 (1.77) | 4.37 (1.71) |
| TRUST IN BROAD INSTITUTIONS |  |  |  |  |  |  |  |
| MEDIA: How much faith do you have in the media? | 1 = very little faith, 7 = a great deal of faith | 3.28 (1.70) | 3.48 (1.67) | 3.42 (1.76) | 3.36 (1.71) | 3.22 (1.71) | 3.19 (1.75) |
| LOCAL GOVERNMENT: How much faith do you have in your local government? | 1 = very little faith, 7 = a great deal of faith | 4.15 (1.61) | 4.34 (1.64) | 4.23 (1.70) | 4.09 (1.65) | 3.97 (1.68) | 3.87 (1.66) |
| FEDERAL GOVERNMENT: How much faith do you have in the federal government? | 1 = very little faith, 7 = a great deal of faith | 3.38 (1.73) | 3.18 (1.76) | 3.00 (1.76) | 2.95 (1.73) | 2.77 (1.75) | 2.70 (1.65) |
| **Demographic Measures** | | | | | | | |
| What is your age? | numeric response | 38.48 (12.21) | 39.93 (12.54) | 40.59 (12.49) | 40.80 (12.43) | 40.14 (12.47) | 40.33 (12.39) |
| What is your gender identity? (proportion female)* | Female, Male, or Other | 45% | 46% | 46% | 46% | 46% | 47% |
| To what extent do you identify with the Republican vs. Democratic party? | 1 = strongly Republican, 7 = strongly Democratic | 3.98 (1.49) | 3.94 (1.54) | 4.00 (1.53) | 3.98 (1.55) | 3.99 (1.57) | 4.00 (1.55) |
| Subjective socioeconomic status | 1-10 | 4.95 (1.58) | 4.90 (1.59) | 4.94 (1.54) | 4.96 (1.58) | 4.94 (1.58) | 4.93 (1.60) |

S2 Table shows mean values, with standard deviation in parentheses, for each measure and construct by wave, except gender showing proportion identifying as female. Outcomes analyzed in the text are highlighted in gray. All constructs are labeled as such, and the measures they are composed of are indented underneath. ® indicates reverse coded measures. W = wave.

*Participants who did not identify as either male or female constituted around .65% of the sample in each wave.
