# Supplementary material for: COVID-19 and vaccine hesitancy: A longitudinal study
Source: PLoS One. 2021 Apr 16;16(4):e0250123. doi: 10.1371/journal.pone.0250123 (PMC8051771; doi:10.1371/journal.pone.0250123)
Supplement: S3 Table — (DOCX) [file pone.0250123.s005.docx]

**S3 Table. Summary table of measures excluded from the text.**

| **Measures** | **Scale Anchors** | **Mar (W1)** | **Apr (W2)** | **May (W3)** | **Jun (W4)** | **Jul (W5)** | **Aug (W6)** |
| --- | --- | --- | --- | --- | --- | --- | --- |
| To what extent did you feel each of the following today? - Fearful | 1 = not at all, 7 = very much so | X | X | X | X | X | X |
| To what extent did you feel each of the following today? - Anxious | 1 = not at all, 7 = very much so | X | X | X | X | X | X |
| To what extent did you feel each of the following today? - Worthlessness | 1 = not at all, 7 = very much so | X | X | X | X | X | X |
| To what extent did you feel each of the following today? - Nervousness | 1 = not at all, 7 = very much so | X | X | X | X | X | X |
| To what extent did you feel each of the following today? - Sadness | 1 = not at all, 7 = very much so | X | X | X | X | X | X |
| Over the last several days, have you been continually worried or anxious about a number of events or activities in your daily life? | 0 = no, 1 = yes |  |  | X | X | X | X |
| Handwashing: On average, how many times per day did you wash your hands this past week? | 0-10, more than 10 | X | X | X | X | X | X |
| I am avoiding contact with others due to Coronavirus concerns | 1 = strongly disagree, 7 = strongly agree | X | X | X | X | X | X |
| I am avoiding crowded places due to Coronavirus concerns | 1 = strongly disagree, 7 = strongly agree | X | X | X | X | X | X |
| I am making purchases with Coronavirus in mind | 1 = strongly disagree, 7 = strongly agree | X | X | X | X | X | X |
| Since mid-March, other people's attitudes and behaviors in response to the COVID-19 pandemic may have changed or stayed the same. Whose attitudes and behaviors do you believe have changed more, Republicans or Democrats? - Concerns about COVID-19 | 1 = Republicans changed much more, 4 = They have changed equally, 7 = Democrats changed much more |  |  |  |  |  | X |
| Since mid-March, other people's attitudes and behaviors in response to the COVID-19 pandemic may have changed or stayed the same. Whose attitudes and behaviors do you believe have changed more, Republicans or Democrats? - Beliefs about healthcare | 1 = Republicans changed much more, 4 = They have changed equally, 7 = Democrats changed much more |  |  |  |  |  | X |
| Since mid-March, other people's attitudes and behaviors in response to the COVID-19 pandemic may have changed or stayed the same. Whose attitudes and behaviors do you believe have changed more, Republicans or Democrats? - Attitudes toward vaccinations in general | 1 = Republicans changed much more, 4 = They have changed equally, 7 = Democrats changed much more |  |  |  |  |  | X |
| Since mid-March, other people's attitudes and behaviors in response to the COVID-19 pandemic may have changed or stayed the same. Whose attitudes and behaviors do you believe have changed more, Republicans or Democrats? - Attitudes toward the COVID-19 vaccine | 1 = Republicans changed much more, 4 = They have changed equally, 7 = Democrats changed much more |  |  |  |  |  | X |
| Since mid-March, other people's attitudes and behaviors in response to the COVID-19 pandemic may have changed or stayed the same. Whose attitudes and behaviors do you believe have changed more, Republicans or Democrats? - Social distancing | 1 = Republicans changed much more, 4 = They have changed equally, 7 = Democrats changed much more |  |  |  |  |  | X |
| Since mid-March, other people's attitudes and behaviors in response to the COVID-19 pandemic may have changed or stayed the same. Whose attitudes and behaviors do you believe have changed more, Republicans or Democrats? - Faith in local government | 1 = Republicans changed much more, 4 = They have changed equally, 7 = Democrats changed much more |  |  |  |  |  | X |
| Since mid-March, other people's attitudes and behaviors in response to the COVID-19 pandemic may have changed or stayed the same. Whose attitudes and behaviors do you believe have changed more, Republicans or Democrats? - Faith in federal government | 1 = Republicans changed much more, 4 = They have changed equally, 7 = Democrats changed much more |  |  |  |  |  | X |
| Since mid-March, other people's attitudes and behaviors in response to the COVID-19 pandemic may have changed or stayed the same. Whose attitudes and behaviors do you believe have changed more, Republicans or Democrats? - Faith in the media | 1 = Republicans changed much more, 4 = They have changed equally, 7 = Democrats changed much more |  |  |  |  |  | X |
| Since mid-March, your attitudes and behaviors in response to the COVID-19 pandemic may have changed or stayed the same. To what extent do you believe that your attitudes and behaviors (described below) either stayed the same or changed? - Concerns about COVID-19 | 1 = Stayed the same, 7 = Changed drastically |  |  |  |  |  | X |
| Since mid-March, your attitudes and behaviors in response to the COVID-19 pandemic may have changed or stayed the same. To what extent do you believe that your attitudes and behaviors (described below) either stayed the same or changed? - Beliefs about healthcare | 1 = Stayed the same, 7 = Changed drastically |  |  |  |  |  | X |
| Since mid-March, your attitudes and behaviors in response to the COVID-19 pandemic may have changed or stayed the same. To what extent do you believe that your attitudes and behaviors (described below) either stayed the same or changed? - Attitudes toward vaccinations in general | 1 = Stayed the same, 7 = Changed drastically |  |  |  |  |  | X |
| Since mid-March, your attitudes and behaviors in response to the COVID-19 pandemic may have changed or stayed the same. To what extent do you believe that your attitudes and behaviors (described below) either stayed the same or changed? - Attitudes toward the COVID-19 vaccine | 1 = Stayed the same, 7 = Changed drastically |  |  |  |  |  | X |
| Since mid-March, your attitudes and behaviors in response to the COVID-19 pandemic may have changed or stayed the same. To what extent do you believe that your attitudes and behaviors (described below) either stayed the same or changed? - Social distancing | 1 = Stayed the same, 7 = Changed drastically |  |  |  |  |  | X |
| Since mid-March, your attitudes and behaviors in response to the COVID-19 pandemic may have changed or stayed the same. To what extent do you believe that your attitudes and behaviors (described below) either stayed the same or changed? - Faith in local government | 1 = Stayed the same, 7 = Changed drastically |  |  |  |  |  | X |
| Since mid-March, your attitudes and behaviors in response to the COVID-19 pandemic may have changed or stayed the same. To what extent do you believe that your attitudes and behaviors (described below) either stayed the same or changed? - Faith in federal government | 1 = Stayed the same, 7 = Changed drastically |  |  |  |  |  | X |
| Since mid-March, your attitudes and behaviors in response to the COVID-19 pandemic may have changed or stayed the same. To what extent do you believe that your attitudes and behaviors (described below) either stayed the same or changed? - Faith in the media | 1 = Stayed the same, 7 = Changed drastically |  |  |  |  |  | X |
| I think the Black Lives Matter movement is important | 1 = strongly disagree, 7 = strongly agree |  |  |  | X | X | X |
| I think it is important for people to protest police brutality | 1 = strongly disagree, 7 = strongly agree |  |  |  | X | X | X |
| I think that the United States needs to work on reducing racial biases | 1 = strongly disagree, 7 = strongly agree |  |  |  | X | X | X |
| I do not think that people in the United States are afforded equal rights | 1 = strongly disagree, 7 = strongly agree |  |  |  | X | X | X |
| When there is a vaccine available for the Coronavirus, I would want to get it.* | 1 = strongly disagree, 7 = strongly agree |  | X | X | X | X | X |
| Do you have children? | 0 = no, 1 = yes |  |  |  |  |  | X |
| What is the age (in years) of your youngest child? | numeric response |  |  |  |  |  | X |
| I have found ways to cope with this disruption | 1 = not at all, 7 = very much so |  |  |  |  | X | X |
| It has been difficult to cope with this disruption | 1 = not at all, 7 = very much so |  |  |  |  | X | X |
| Over the last several days, have you felt depressed or sad much of the time? | 0 = no, 1 = yes |  |  | X | X | X | X |
| Dictator Game | numeric response | X | X | X | X | X | X |
| How much faith do you have in scientific institutions? | 1 = very little faith, 7 = a great deal of faith |  |  |  | X | X | X |
| Have you experienced a significant disruption in your personal finances due to the COVID-19 pandemic? | 0 = no, 1 = yes |  |  | X | X | X | X |
| Have you gotten the flu vaccine at least once in the past two years? | 0 = no, 1 = yes | X | X | X | X | X | X |
| Employers should provide paid sick leave to their employees | 1 = strongly disagree, 7 = strongly agree | X | X | X | X | X | X |
| Access to health care is a fundamental human right | 1 = strongly disagree, 7 = strongly agree | X | X | X | X | X | X |
| The government should guarantee health care access to all citizens | 1 = strongly disagree, 7 = strongly agree | X | X | X | X | X | X |
| The COVID-19 Pandemic has created an impossibly harsh reality for many people. | 1 = strongly disagree, 7 = strongly agree |  |  |  |  |  | X |
| Have you lost your health insurance during the pandemic? | 0 = no, 1 = yes |  |  |  |  | X | X |
| Did you lose your job due to the COVID-19 pandemic? | 0 = no, 1 = yes |  |  |  |  | X | X |
| To what extent would you consider each of these factors in your decision of whether to get the Coronavirus vaccine? - The influence of God/My religion | 1 = not at all, 7 = very much | X | X | X | X | X | X |
| To what extent would you consider each of these factors in your decision of whether to get the Coronavirus vaccine? - I am concerned about the effect of the Coronavirus on me | 1 = not at all, 7 = very much | X | X | X | X | X | X |
| To what extent would you consider each of these factors in your decision of whether to get the Coronavirus vaccine? - I want to protect close others who are at risk | 1 = not at all, 7 = very much | X | X | X | X | X | X |
| To what extent would you consider each of these factors in your decision of whether to get the Coronavirus vaccine? - It feels like the only way for life to get back to normal | 1 = not at all, 7 = very much | X | X | X | X | X | X |
| To what extent would you consider each of these factors in your decision of whether to get the Coronavirus vaccine? - I want to reduce my anxiety about this virus | 1 = not at all, 7 = very much | X | X | X | X | X | X |
| Do you consider yourself to be part of one or more minority groups in the United States? | 0 = no, 1 = yes |  |  |  | X | X |  |
| Have you found another income source since losing your job? | 0 = no, 1 = yes |  |  |  |  | X | X |
| Have you tested positive for Coronavirus? | 0 = no, 1 = yes | X | X | X | X | X | X |
| Has anyone close to you tested positive for Coronavirus? | 0 = no, 1 = yes | X | X | X | X | X | X |
| How many people in your life have tested positive for Coronavirus? | numeric response |  | X | X | X | X | X |
| I am satisfied with my social life | 1 = strongly disagree, 7 = strongly agree | X | X | X | X | X | X |
| I am satisfied with my friend network | 1 = strongly disagree, 7 = strongly agree | X | X | X | X | X | X |
| I am satisfied with my abilities | 1 = strongly disagree, 7 = strongly agree | X | X | X | X | X | X |
| I am happy overall | 1 = strongly disagree, 7 = strongly agree | X | X | X | X | X | X |
| I am a good friend | 1 = strongly disagree, 7 = strongly agree | X | X | X | X | X | X |
| I am satisfied with my achievements | 1 = strongly disagree, 7 = strongly agree | X | X | X | X | X | X |
| Other people enjoy being around me | 1 = strongly disagree, 7 = strongly agree | X | X | X | X | X | X |
| Trust Game | numeric response | X | X | X | X | X | X |
| What is your zipcode? | text response | X |  |  |  |  |  |
| Any other comments about what is happening surrounding Coronavirus in your family, community, in the United States or in the World? | text response | X | X | X |  |  |  |
| You stated that you experienced a significant disruption in your personal finances due to the COVID- 19 pandemic. When did this disruption occur? | March; April; May; June; July; August |  |  |  |  | X | X |
| What is the highest level of school you have completed or the highest degree you have received? | Less than high school degree; High school graduate (high school diploma or equivalent including GED); Some college but no degree; Associate degree in college (2-year); Bachelor's degree in college (4-year); Master's degree; Doctoral degree; Professional degree (JD, MD) |  |  |  |  | X |  |
| When asked whether you plan on getting the flu vaccine next year (1=Definitely no, 7= Definitely yes) you indicated a {numeric response}. In a sentence, please explain your answer. | text response |  |  |  |  | X |  |
| Please indicate you household income for last year before taxes (your best guess). | Less than $9,999; $10,000 to $19,999; $20,000 to $29,999; $30,000 to $39,999; $40,000 to $49,999; $50,000 to $59,999; $60,000 to $69,999; $70,000 to $79,999; $80,000 to $89,999; $90,000 to $99,999; $100,000 to $149,999; $150,000 or more |  |  |  |  | X |  |
| Which of the following industries most closely matches the one in which you are employed (or were most recently employed)? | Forestry, fishing, hunting or agriculture support; Real estate or rental and leasing; Mining; Professional, scientific, or technical services; Utilities; Management of companies or enterprises; Construction; Admin, support, waste management or remediation services; Manufacturing; Educational services; Wholesale trade; Health care or social assistance; Retail trade; Arts, entertainment or recreation; Transportation or warehousing; Accommodation or food services; Information; Finance or insurance; I am a full-time student; I am retired; None of the above |  |  |  |  | X | X |
| You stated that you consider yourself to be part of one or more minority groups. Which of these categories do these groups fall into? | Race; Sexual preference; Religion; Gender identity; Other (text response) |  |  |  | X | X |  |
| Which of these news sources do you regularly check (about once a day)? | ABC News; CBS News; CNN; Facebook or Instagram; Fox News; HuffPost; Los Angeles Times; MSNBC; NBC News; NPR; The New York Times; The Wall Street Journal; The Washington Post; Twitter; USA Today; Other (text response) |  |  |  | X | X | X |
| What is your racial or ethnic identification? (Mark all that apply) | American Indian or Alaska Native; Asian; Black or African American; Native Hawaiian or Pacific Islander; White; Hispanic, Latino, or Spanish origin; Not listed (please specify) (text response) |  |  |  | X | X |  |
| Which of the following do you believe is true? The worst of the COVID-19 pandemic is: | behind us; now; yet to come |  |  |  |  |  | X |

Additional measures collected, excluded from the text. Xs indicate that the measure was collected on the corresponding wave of the survey. W = wave.

* This item was added in wave 2 due to the concern that participants responding to the item “When there is a vaccine available for the Coronavirus, I will get it” may have interest in the vaccine, but not believe they will have access to it. However, these two measures were highly correlated (*r* > .94).
