# Supplementary material for: COVID-19 and vaccine hesitancy: A longitudinal study
Source: PLoS One. 2021 Apr 16;16(4):e0250123. doi: 10.1371/journal.pone.0250123 (PMC8051771; doi:10.1371/journal.pone.0250123)
Supplement: S4 Table — (DOCX) [file pone.0250123.s006.docx]

**S4 Table. Regression results.**

|  | **COVID-19 Vaccination Attitudes** | **General Vaccination Attitudes** | **Flu Shot Intentions** |
| --- | --- | --- | --- |
| **wave** | -0.0446*** (0.0102) [-0.0645; -0.0247] | -0.0179*** (0.00459) [-0.0269; -0.0089] | -0.0184 (0.00941) [-0.0369; 1.13e-05] |
| **wave x Democrat** |  |  |  |
| **wave x Republican** |  |  |  |
| **wave x Age** |  |  |  |
| **wave x SES** |  |  |  |
| **wave x Democrat x**  **political affiliation strength** |  |  |  |
| **wave x Republican x**  **political affiliation strength** |  |  |  |
| **Observations** | 4,335 | 4,295 | 4,309 |
| **R^2^** | 0.85391 | 0.92065 | 0.90706 |
| **Within R^2^** | 0.01111 | 0.00659 | 0.00177 |

|  | **COVID-19 Vaccination Attitudes** | **General Vaccination Attitudes** | **Flu Shot Intentions** | **Flu Shot Intentions** | **Perceived Threat of COVID-19** | **Perceived Threat of COVID-19** |
| --- | --- | --- | --- | --- | --- | --- |
| **wave** |  |  |  |  |  |  |
| **wave x Democrat** | 0.018 (0.0422) [-0.0648; 0.101] | -0.0413* (0.0189) [-0.0784; -0.00428] | 0.00662 (0.0378) [-0.0675; 0.0807] | 0.0242 (0.0404) [-0.0551; 0.103] | 0.0776* (0.0364) [0.0063; 0.149] | 0.0512 (0.0393) [-0.0259; 0.128] |
| **wave x Republican** | -0.0919* (0.046) [-0.182; -0.00172] | -0.0853*** (0.0232) [-0.131; -0.0398] | -0.115** (0.041) [-0.196; -0.0348] | -0.0815* (0.0407) [-0.161; -0.00173] | -0.00778 (0.0383) [-0.0828; 0.0673] | -0.0104 (0.0413) [-0.0913; 0.0705] |
| **wave x Age** | -0.000897 (0.000775) [-0.00242; 0.000623] | 0.000562 (0.00038) [-0.000182; 0.00131] | 0.00023 (0.000664) [-0.00107; 0.00153] | 0.000368 (0.000664) [-0.000934; 0.00167] | 0.00109 (0.000644) [-0.000168; 0.00236] | 0.00101 (0.000644) [-0.000249; 0.00228] |
| **wave x SES** | 0.00316 (0.00633) [-0.00926; 0.0156] | 0.00365 (0.00273) [-0.0017; 0.00901] | 0.00241 (0.0058) [-0.00895; 0.0138] | 0.0015 (0.00579) [-0.00985; 0.0128] | -0.0179*** (0.00527) [-0.0282; -0.00756] | -0.0179*** (0.00526) [-0.0282; -0.0076] |
| **wave x Democrat x political affiliation strength** |  |  |  | -0.0188 (0.0145) [-0.0471; 0.00956] |  | 0.0299* (0.0128) [0.00493; 0.0549] |
| **wave x Republican x political affiliation strength** |  |  |  | -0.0525* (0.0237) [-0.099; -0.00596] |  | 0.00934 (0.0186) [-0.0272; 0.0459] |
| **Observations** | 3,964 | 3,935 | 3,952 | 3,952 | 3,958 | 3,958 |
| **R^2^** | 0.85298 | 0.91884 | 0.90746 | 0.90784 | 0.79896 | 0.79953 |
| **Within R^2^** | 0.02913 | 0.01649 | 0.02048 | 0.02445 | 0.02143 | 0.0242 |

|  | **Trust in Media** | **Trust in Local Government** | **Trust in Federal Government** |
| --- | --- | --- | --- |
| **wave** |  |  |  |
| **wave x Democrat** | 0.0202 (0.036) [-0.0505; 0.0908] | -0.0574 (0.0437) [-0.143; 0.0282] | -0.0806* (0.0383) [-0.156; -0.00544] |
| **wave x Republican** | -0.0511 (0.0401) [-0.13; 0.0276] | -0.115* (0.0472) [-0.207; -0.0222] | -0.0987* (0.0439) [-0.185; -0.0126] |
| **wave x Age** | -0.000339 (0.00066) [-0.00163; 0.000954] | -0.000708 (0.000724) [-0.00213; 0.00071] | -0.000123 (0.000744) [-0.00158; 0.00133] |
| **wave x SES** | -0.00439 (0.0049) [-0.014; 0.0052] | 0.00471 (0.006) [-0.00705; 0.0165] | -0.00823 (0.00567) [-0.0193; 0.0029] |
| **wave x Democrat x**  **political affiliation strength** |  |  |  |
| **wave x Republican x**  **political affiliation strength** |  |  |  |
| **Observations** | 3,960 | 3,960 | 3,960 |
| **R^2^** | 0.80638 | 0.75761 | 0.80298 |
| **Within R^2^** | 0.01614 | 0.03505 | 0.08233 |

Significance codes: *** P < .001, ** P < .01, * P < .05.

Full regression results of regressions described in the text. Standard errors are in parentheses, and 95% confidence intervals are in square brackets. All models include individual-level fixed effects, and the standard errors are clustered at the individual level.
