# Supplementary material for: COVID-19 and vaccine hesitancy: A longitudinal study
Source: PLoS One. 2021 Apr 16;16(4):e0250123. doi: 10.1371/journal.pone.0250123 (PMC8051771; doi:10.1371/journal.pone.0250123)
Supplement: S5 Table — (DOCX) [file pone.0250123.s007.docx]

**S5 Table. Outcome measures by political party affiliation.**

| **Measure** | **Wave** | **Democrats** | **Republicans** | **P-value** |
| --- | --- | --- | --- | --- |
| COVID-19 Vaccination Attitudes | 1 | 5.39 (0.062) | 4.57 (0.091) | <.001 |
|  | 2 | 5.63 (0.072) | 4.59 (0.111) | <.001 |
|  | 3 | 5.57 (0.08) | 4.21 (0.128) | <.001 |
|  | 4 | 5.53 (0.085) | 4.11 (0.128) | <.001 |
|  | 5 | 5.58 (0.083) | 4.21 (0.127) | <.001 |
|  | 6 | 5.39 (0.086) | 3.92 (0.124) | <.001 |
| General Vaccination Attitudes | 1 | 5.83 (0.047) | 5.17 (0.069) | <.001 |
|  | 2 | 5.83 (0.055) | 5.11 (0.079) | <.001 |
|  | 3 | 5.84 (0.056) | 5.05 (0.085) | <.001 |
|  | 4 | 5.83 (0.062) | 5.07 (0.09) | <.001 |
|  | 5 | 5.91 (0.057) | 5.01 (0.09) | <.001 |
|  | 6 | 5.86 (0.058) | 4.9 (0.088) | <.001 |
| Flu Shot Intentions | 1 | 4.84 (0.094) | 4.35 (0.123) | 0.002 |
|  | 2 | 4.92 (0.107) | 4.33 (0.145) | <.001 |
|  | 3 | 5 (0.113) | 4.12 (0.159) | <.001 |
|  | 4 | 4.97 (0.118) | 3.93 (0.162) | <.001 |
|  | 5 | 5.07 (0.115) | 4.01 (0.16) | <.001 |
|  | 6 | 5.01 (0.112) | 3.86 (0.161) | <.001 |
| Perceived Threat of COVID-19 | 1 | 4.26 (0.05) | 3.9 (0.072) | <.001 |
|  | 2 | 4.46 (0.055) | 4.09 (0.086) | <.001 |
|  | 3 | 4.36 (0.063) | 3.71 (0.098) | <.001 |
|  | 4 | 4.29 (0.066) | 3.54 (0.099) | <.001 |
|  | 5 | 4.49 (0.062) | 3.78 (0.098) | <.001 |
|  | 6 | 4.47 (0.062) | 3.7 (0.097) | <.001 |
| Trust: Media | 1 | 3.61 (0.067) | 2.73 (0.085) | <.001 |
|  | 2 | 3.88 (0.072) | 2.82 (0.097) | <.001 |
|  | 3 | 3.94 (0.081) | 2.55 (0.103) | <.001 |
|  | 4 | 3.92 (0.081) | 2.45 (0.101) | <.001 |
|  | 5 | 3.75 (0.078) | 2.36 (0.101) | <.001 |
|  | 6 | 3.73 (0.08) | 2.29 (0.1) | <.001 |
| Trust: Local Government | 1 | 4.07 (0.064) | 4.28 (0.083) | 0.045 |
|  | 2 | 4.39 (0.074) | 4.26 (0.1) | 0.267 |
|  | 3 | 4.37 (0.082) | 4 (0.111) | 0.007 |
|  | 4 | 4.2 (0.084) | 3.91 (0.11) | 0.039 |
|  | 5 | 4.02 (0.084) | 3.88 (0.106) | 0.281 |
|  | 6 | 3.94 (0.082) | 3.76 (0.105) | 0.172 |
| Trust: Federal Government | 1 | 2.96 (0.067) | 4.08 (0.082) | <.001 |
|  | 2 | 2.58 (0.071) | 4.16 (0.099) | <.001 |
|  | 3 | 2.52 (0.078) | 3.82 (0.111) | <.001 |
|  | 4 | 2.49 (0.082) | 3.71 (0.111) | <.001 |
|  | 5 | 2.28 (0.079) | 3.57 (0.109) | <.001 |
|  | 6 | 2.3 (0.075) | 3.37 (0.104) | <.001 |

S4 Table shows the average value, with the corresponding standard error in parentheses, for every wave of each outcome measure, by political party affiliation. P-values are results from an ANOVA, comparing Democrats and Republicans in each wave for the corresponding outcome measure.
