# Supplementary material for: COVID-19 and vaccine hesitancy: A longitudinal study
Source: PLoS One. 2021 Apr 16;16(4):e0250123. doi: 10.1371/journal.pone.0250123 (PMC8051771; doi:10.1371/journal.pone.0250123)
Supplement: S6 Table — (DOCX) [file pone.0250123.s008.docx]

**S6 Table. Summary of news sources.**

|  | **Democrats** | **Republicans** | **Overall** |
| --- | --- | --- | --- |
| CNN | 47% | 24% | 38% |
| Facebook or Instagram | 28% | 35% | 31% |
| The New York Times | 34% | 12% | 25% |
| Fox News | 8% | 49% | 24% |
| Twitter | 25% | 19% | 23% |
| NPR | 28% | 9% | 21% |
| The Washington Post | 24% | 9% | 18% |
| Other | 15% | 22% | 18% |
| NBC News | 19% | 13% | 17% |
| ABC News | 19% | 13% | 17% |
| MSNBC | 19% | 10% | 16% |
| CBS News | 15% | 13% | 14% |
| The Wall Street Journal | 10% | 16% | 13% |
| USA Today | 11% | 7% | 9% |
| HuffPost | 10% | 3% | 7% |
| Los Angeles Times | 3% | 3% | 3% |

The popularity of news sources among respondents: The proportion of participants indicating they regularly check the news source. Sorted by overall popularity. This question was introduced in wave 4 and subsequently remained in every wave of the survey; the response to the first wave in which a participant responded to the question was used for the table.
