# Supplementary material for: COVID-19 and vaccine hesitancy: A longitudinal study
Source: PLoS One. 2021 Apr 16;16(4):e0250123. doi: 10.1371/journal.pone.0250123 (PMC8051771; doi:10.1371/journal.pone.0250123)
Supplement: Attachment — Submitted filename: Response to Reviewers.docx [file pone.0250123.s009.docx]

February 3, 2021

*COVID-19 and Vaccine Hesitancy: A Longitudinal Study*

Dear Dr. Capraro,

Thank you for the opportunity to revise our manuscript, titled “*COVID-19 and Vaccine Hesitancy: A Longitudinal Study*” for resubmission to *PLOS ONE*. We greatly appreciated the constructive feedback we received. Below we have outlined our response to each point that you and the reviewer raised in the review.

*AE: I have recently published a paper where we look at the correlation between risk perception and intentions to wear a face mask. The results are in line with your study. I think it could be interesting to relate these works.*

**Thank you for bringing this paper to our attention. We incorporated it into the general discussion, on lines 329-331.**

*R: I find a bit awkward the choice of the authors of introducing parts that should pertain to the discussion of the results in the introduction (i.e. lines 62-65 and 92-100).*

**Thank you for your feedback on the layout of the introduction. We streamlined the introduction, as per your suggestion, and removed lines 62-65. We revised lines 92-100 as well, which constitute the last few lines of the introduction and transition to the empirical section. We believe that these lines provide an important preview of the results which may be helpful to readers seeking a quick summary. However, if you still feel that this way of introducing the results is inappropriate, we will change it.**

*R: The same goes for the results section: in this section, the authors included not only the results, but also a (a bit confused, in my opinion) explanation of data analyses. I recommend the authors to re-organize this section, adding a paragraph in "methods" to explain their analyses plan before describing results: an example (but not exhaustive) are lines from 148 to 153, as these are methods and not results. Another example are lines 169-172. Re-organizing these sections will greatly increase readability and clarity.*

**Based on your feedback, we have made changes throughout the methods, results, and discussions sections to increase clarity. For example, as the reviewer suggested we moved lines 148-153 and 169-172 to the methods section (now 140-145 and 119-125, respectively). We also included a paragraph describing our analysis plan. We believe that these edits have improved the manuscript. Thank you for the suggestion.**

*R: I was expecting an approach based on the analysis of variance (ANOVA), to address means differences within waves and between different groups. So, I'm not really sure that the approach adopted by the authors is the most suitable. However, I expect that the aforementioned re-organization of the methods and results sections will help (me and the future readers) to understand the authors' choices, and the authors to justify the methods they adopted.*

**We re-organized the methods and results section, and hope they are now clearer. In the newly added analysis plan paragraph (lines 140-148), we provided additional justification for the fixed-effects regression model we used to analyze our data. Furthermore, based on your suggestion, we added an ANOVA table with all results to the manuscript (lines 174-176, table S4).**

*R: Moreover, given sample size and the number of tested hypotheses, I would like the authors to address the fact that some of their "significances" were rather marginal (e.g. see p-value=.046 considered significant at line 188). I feel like the authors should address this by either adopting a more conservative value of p (instead of the usual p=.05), or by adding some note of caution in the discussion for those results that are only marginally significant. On the same page, I would like the authors to add effect sizes were applicable, e.g. Cohen's d (or similar) when reporting t-tests.*

**The point about P-values is well taken. We included a sentence (line 145) explicitly pointing out that the P-values reported are not adjusted for multiple testing. Part of the reason we decided not to adjust the P-values is due to the fact that there is not a broad consensus on the correct way to do this. However, all data is available to readers who would like to adjust the P-values in their preferred way. Furthermore, the particular P-value the reviewer mentioned, regarding the time trend for Republicans, is not pertinent to our claim of divergence between Democrats and Republicans over time (which are P < .001 on all the vaccination attitudes and intentions measures). We also added Cohen’s d effect sizes for all t-tests, as suggested, as well as 95% confidence intervals for the effect sizes.**

*R: Finally, one last concern regarding the sample: were any strategies used to check data quality? Unfortunately, using data from panel results sometimes in some participants being "professional respondents": were any countermeasures taken (e.g. screening of multivariate outliers, uncommon response patterns or survey completion times)? If not, this should be address and discussed by the authors in the manuscript.*

**While data from Amazon’s Mechanical Turk has advantages and disadvantages, with extant research examining the reliability and quality of this sample (e.g., Goodman, Cryder, and Cheema 2012), we have confidence in our choice to use this population for our study. We can further use our own data to demonstrate the quality of our sample. One indication that participants are paying attention is that the demographic makeup of our sample is stable over time, indicating that they did not respond to these questions at random. Due to your concerns, we ran an additional robustness check in which we removed participants with completion times below the 10th percentile, corresponding to less than 3 minutes. We found a similar pattern of results, though some coefficients were no longer significant at the .05 level, which is unsurprising given the smaller sample size. These include: overall decline in flu shot intentions (p = .06), Republican’ decline in COVID-19 vaccination attitudes (p = .10), Democrats’ decline in general vaccination attitudes (p = .08), Democrats’ increase in perceived threat of COVID-19 (p = .06). Importantly, the difference in trends between Democrats and Republicans remained significant in all cases. This robustness check and a robustness check that includes only participants who completed all 6 waves of the study can be easily run using our code. We would also argue that if the participant quality were low or if participants were not paying attention, this would add noise to the data, and work against finding significant results, rather than introduce a systematic bias.**

**Reference: Goodman, Joseph K., Cynthia E. Cryder, and Amar Cheema. "Data collection in a flat world: The strengths and weaknesses of Mechanical Turk samples." *Journal of Behavioral Decision Making* 26, no. 3 (2013): 213-224.**

Once again, we very much appreciate the in-depth feedback we received and think that the paper is much improved as a result of these changes. We hope that you will agree and we look forward to receiving your reply.

Sincerely,

Ariel Fridman

PhD Candidate in Marketing, Rady School of Management, UC San Diego

Rachel Gershon

Assistant Professor of Marketing, Rady School of Management, UC San Diego

Ayelet Gneezy

Professor of Behavioral Sciences and Marketing, Rady School of Management, UC San Diego
